# King Abdulaziz University Hospital Capsule dataset: A novel small-bowel endoscopic image repository from Saudi Arabia

**DOI:** 10.1016/j.dib.2024.111093

**Published:** 2024-11-08

**Authors:** Hamza Ghandorh, Hamza H. Bali, Wael M.S. Yafooz, Wadii Boulila, Majid Alsahafi

**Affiliations:** a Department of Computer Science, College of Computer Science and Engineering, Taibah University, Medina, Saudi Arabia; b Division of Gastroenterology, Department of Medicine, King Abdulaziz University, Jeddah, Saudi Arabia; c Robotics and Internet-of-Things Laboratory, Prince Sultan University, Riyadh, Saudi Arabia

**Keywords:** Classification, Small-bowel mucosa, Arteriovenous malformations, Ulcer, Machine learning

## Abstract

Wireless Capsule Endoscopy (WCE) has fundamentally transformed diagnostic methodologies for small-bowel (SB) abnormalities, providing a comprehensive and non-invasive gastrointestinal assessment in contrast to conventional endoscopic procedures. The King Abdulaziz University Hospital Capsule (KAUHC) dataset comprises annotated WCE images specifically curated for Saudi Arabian residents. Comprising 10.7 million frames derived from 157 studies, KAUHC has been classified into Normal, Arteriovenous Malformations, and Ulcer categories. Following the application of specific inclusion and exclusion criteria, 3301 labeled frames derived from WCE 86 studies were identified. Upon admission of patients, the data collection phase of KAUHC was initiated, involving the administration of the OMOM capsule and the use of the OMOM recording device for video documentation. A thorough evaluation of these recordings was undertaken by multiple gastroenterologists to identify any pathological abnormalities. The identified observations are subsequently extracted, categorized, and prepared for validation using Machine Learning (ML) classifiers. The dataset aims not only to address the scarcity of annotated endoscopic imaging resources in the Middle East but also to advance the development of diagnostic tools for ML applications in SB abnormalities and exploratory research on gastrointestinal diseases.

Specifications Table

**Table:** 

Subject	Computer Science\Artificial Intelligence.
Specific subject area	Wireless Capulet Endoscopy for small-bowel abnormalities
Type of data	Image
Data collection	A set of labeled 86 video studies illustrating three categories of SB mucosa (Normal, AVM, Ulcer) were collected between December 2019 and December 2023. These studies were conducted within the Division of Gastroenterology, King Abdulaziz University Hospital, Jeddah, Saudi Arabia, as components of diagnostic gastrointestinal assessments for Saudi Arabian residents. The setup included using the OMOM WCE system (camera capsule, sensors belt, recording device, WCE studies management software, and a computer workstation). The recorded studies and their corresponding frames were examined, categorized, and exported under the supervision of local senior gastroenterologists.
Data source location	Division of Gastroenterology, Department of Medicine, King Abdulaziz University, Jeddah, Saudi Arabia.
Data accessibility	Repository name: KAUHC Dataset Data identification number: 10.17632/h5rb78s3pn.1 Direct URL to data: https://data.mendeley.com/datasets/h5rb78s3pn/1
Related research article	none.

## 1 Value of the Data

The value of the King Abdulaziz University Hospital-Capsule (KAUHC) dataset could be summarized as follows:

• **Lack of prior image datasets:** The KAUHC dataset significantly enhances the availability of publicly accessible endoscopic image repositories, particularly in the Middle East. To the best of our knowledge, no publicly available dataset offers such detailed annotation for small-bowel abnormalities in this region [1]. This claim is supported by a comprehensive review of existing datasets, where similar repositories either focus on different gastrointestinal regions or are limited in scope [2].
• **Reuse potential for machine learning applications:** The annotated structure of the KAUHC dataset is designed for easy integration into machine learning (ML) pipelines. It provides labeled data across three critical categories (Normal, Arteriovenous Malformations, and Ulcers), which allows for diverse ML experimentation and the development of diagnostic tools for real-time endoscopic analysis. This reusable nature stems from the dataset's standardized image format, its clear class annotations, and its applicability across various ML models, including classification, segmentation, and anomaly detection.
• **Interdisciplinary research:** Given the complexity and labor-intensiveness of collecting high-quality endoscopic images, this dataset can be a crucial resource for gastrointestinal (GI) studies, as it overcomes common barriers such as administrative hurdles and high costs associated with gathering large-scale medical image data. This will allow researchers from both clinical and computer science backgrounds to collaborate more effectively.

## 2 Background

Inspecting a significant portion of the SB using traditional endoscopy or device-assisted enteroscopy poses challenges. SB possesses an extensive length of approximately 600 cm and a complex looped-shaped configuration [3]. WCE,^1^ a non-invasive diagnostic tool, was primarily developed to offer diagnostic imaging of SB. The non-interventional nature and straightforwardness of WCE lead most clinicians to utilize it in selecting patients and identifying lesions for interventional endoscopy [4]. Although WCE is considered a primary SB diagnostic method with a high success rate [3,5], the interpretation and diagnosis of WCE patients’ studies is a time-consuming and reader-dependent process. Standard WCE techniques typically capture frames at a rate of two frames per second, sustaining this recording between eight and 12 h, resulting in a substantial volume of 57,600 images [6]. These recordings can be manually viewed by a gastroenterologist as either a video stream or individual frames. Concerns arise among gastroenterologists regarding the potential oversight of anomalies in single frames. It is reported that gastroenterologists, through manual WCE readings, could have a high miss rate of 5.9 % for vascular lesions or 0.5 % for ulcers [6].

## 3 Data Description

This section demonstrates the characteristics of the King Abdulaziz University Hospital Capsule (KAUHC) dataset. This work was carried out with official authorization from the Research Ethics Committee (REC) of KAUH with the Institutional Review Board (IRB) (# 395-22, data: 01\01\2022).

### 3.1 Final data format and file structure

**Fig. 1:**
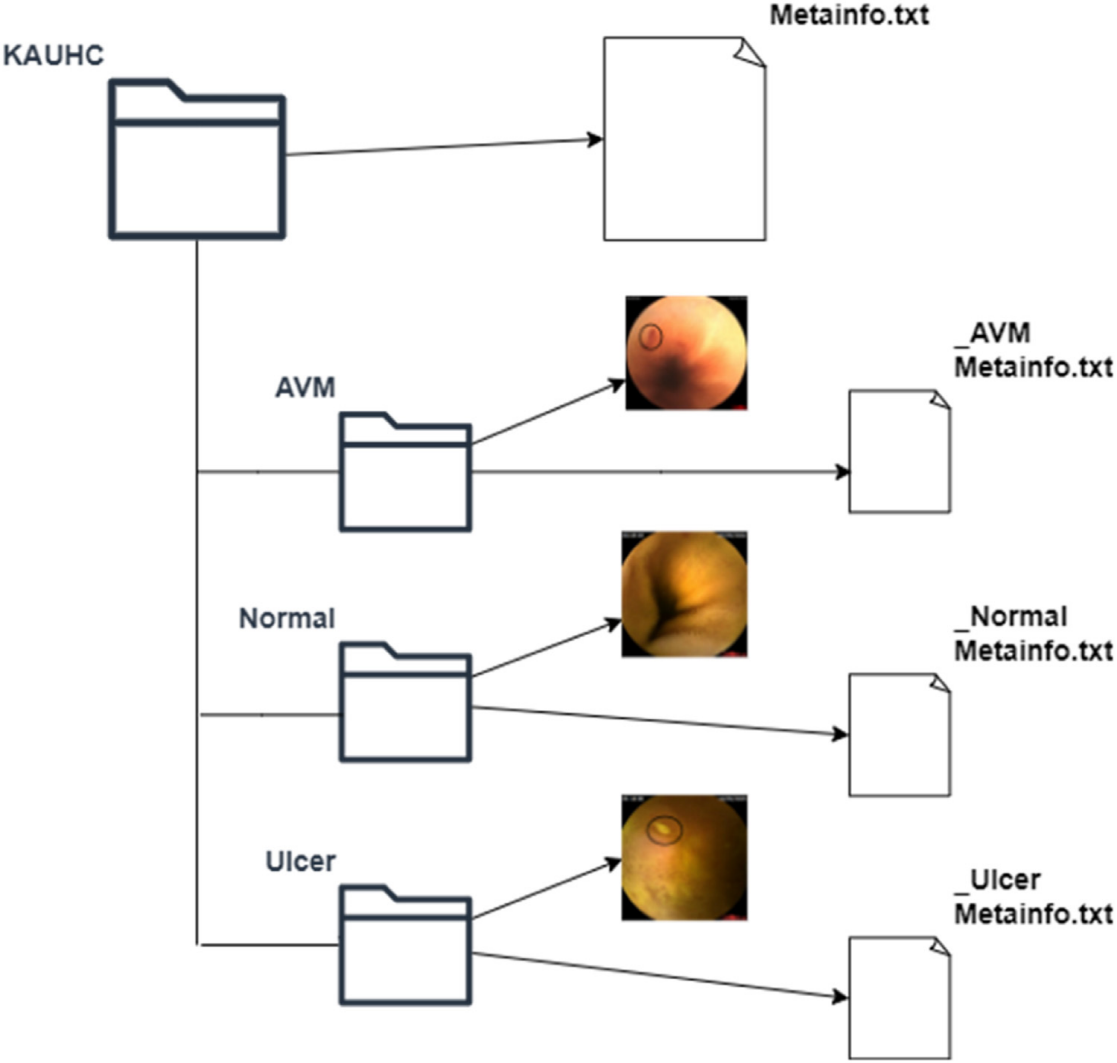
KAUHC dataset folder layout.

The entire dataset can be found in the data directory of the repository. Fig 1 shows how the data directory is organized and arranged into three folders:

• ‘AVM’ frames folder, which holds frames labeled as AVM class.
• ‘Normal’ frames folder, which holds frames labeled as Normal class.
• ‘Ulcer’ frames folder, which holds frames labeled as Ulcer class.

All frames are exported using a raster graphics image file format, namely, ‘.bmp’. The frames filenames contain metadata that describes the corresponding class as follows: “className_timestamp_id.bmp“. A frame exhibits a file size of one megabyte on average, a resolution of 512*×*512 pixels, a bit depth of 32, and remains in an uncompressed state. The image in focus corresponds to an estimated 0.262 mega pixels.

### 3.2 King Abdulaziz University Hospital Capsule (KAUHC)

**Fig. 2:**
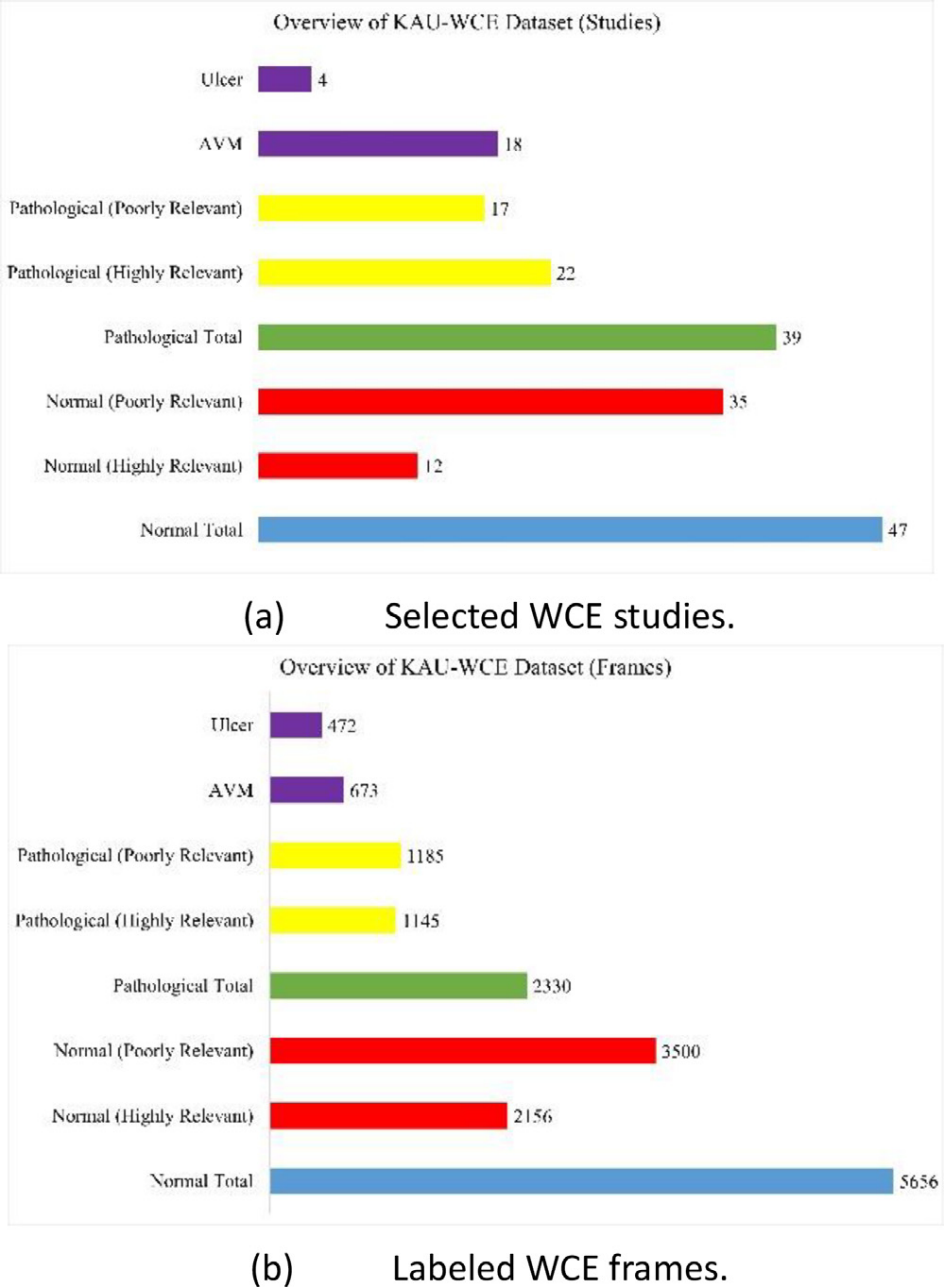
Distribution of the KAUHC dataset in terms of labeled studies and frames.

The distribution of the KAUHC dataset is presented concerning labeled studies and frames, as shown in Fig 2. The total number of unlabeled studies amounted to 157 studies, with an average recording time duration of 9.5 h per recording or study. Each study was captured at a frame rate of 2 frames per second, resulting in an average of 68,400 frames per study. Consequently, the total frames for all studies equated to 10.7 mm frames.

Through gastroenterologists’ censuses, diagnostic investigation reports, and labeling processes, 86 studies were chosen, where 47 studies represent normal small-bowel mucosa (with 5656 frames in total), and 39 studies represent pathological abnormalities (with 2330 frames in total). With the selected normal studies, 12 studies (with 2156 frames in total) were nominated due to their resolution. Within the selected pathological studies, 18 studies with Arteriovenous Malformations (AVM) pathology (with 673 labeled frames) and four studies with Ulcer pathology (with 472 labeled frames) were nominated due to their resolution. The total number of nominated frames in the dataset is 3301, while the entire size of the curated dataset is 3.19 GB.

### 3.3 Classes description

**Fig. 3:**
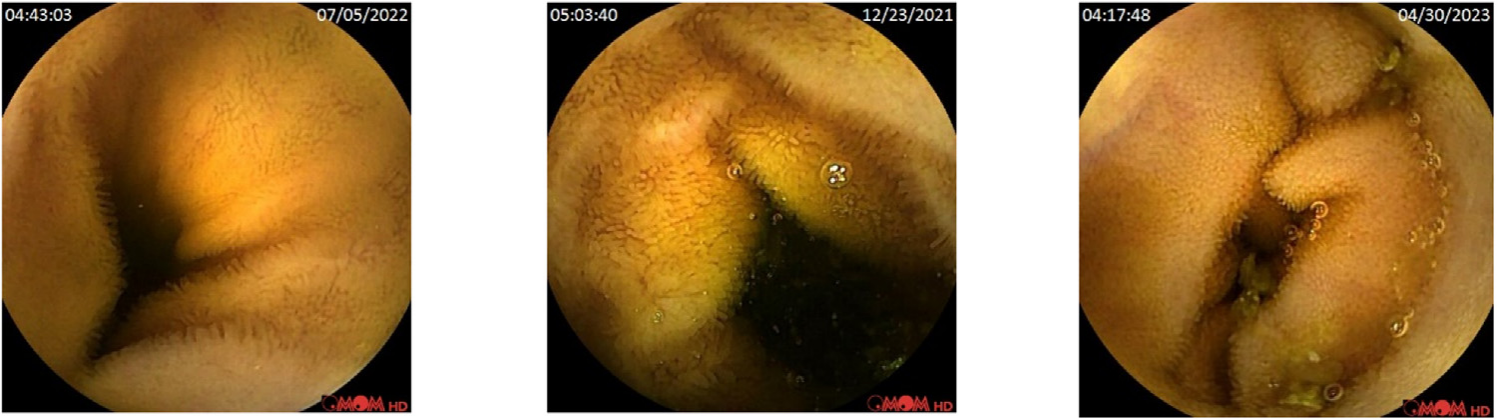
Sample of Normal frames, which are located in the ‘Normal’ folder in the KAUHC dataset.

This section highlights the research findings, detailing the pathological abnormalities observed in this paper. Among normal frames, two main pathological abnormalities were found, namely, AVM and Ulcers. Normal, known as healthy small-bowel mucosa, is a layer of mucous membrane within the SB region in the GI tract. Fig 3 shows a sample of selected normal frames within the dataset, which are located in the ‘Normal’ folder.

**Fig. 4:**
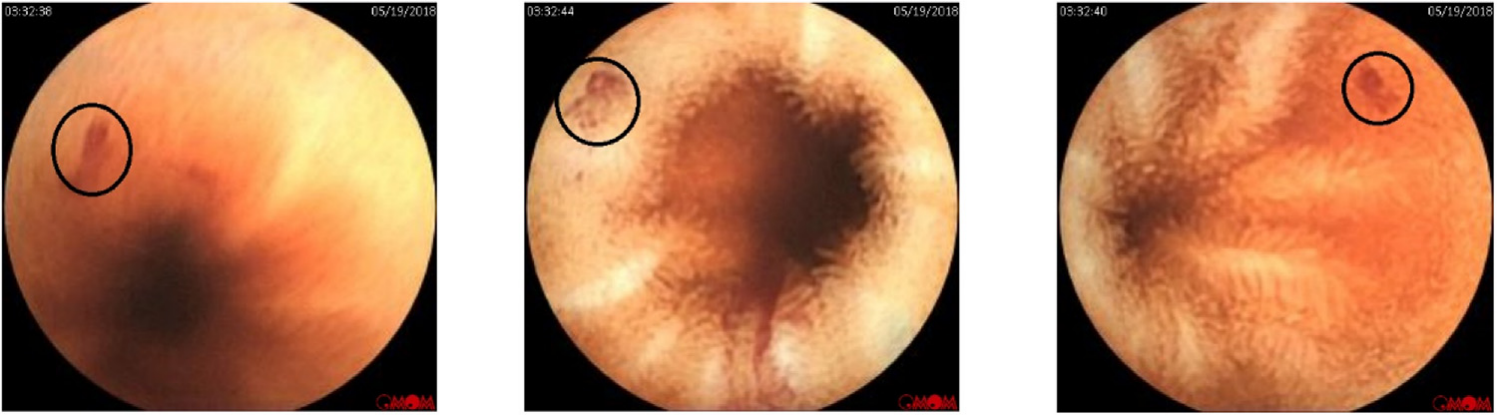
Sample of labeled AVM frames located in the ‘AVM’ folder in the KAUHC dataset.

Arteriovenous Malformations (AVMs), known as angioectasias or angiodysplasias, are abnormal blood vessels in the wall of the GI tract. These abnormal blood vessels are an important vascular cause of gastrointestinal bleeding [7]. Fig 4 shows a sample of selected AVM frames within the dataset, which are located in the ‘AVM’ folder.

**Fig. 5:**
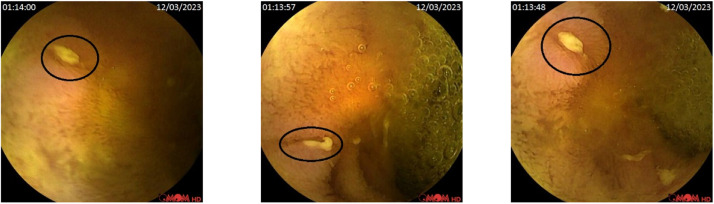
Sample of labeled Ulcer frames, which are located in the ‘Ulcer’ folder in the KAUHC dataset.

An ulcer is a loss of all epithelial cell layers extending to the submucosa. An inflammation carried on by several conditions, inflammatory bowel disease, infection, or drug-induced (e.g., NSAID), is the cause of both erosion and ulcers [8]. Fig 5 shows a sample of selected ulcer frames within the dataset, which are located in the ‘Ulcer’ folder.

### 3.4 Dataset statistics

**Fig. 6:**
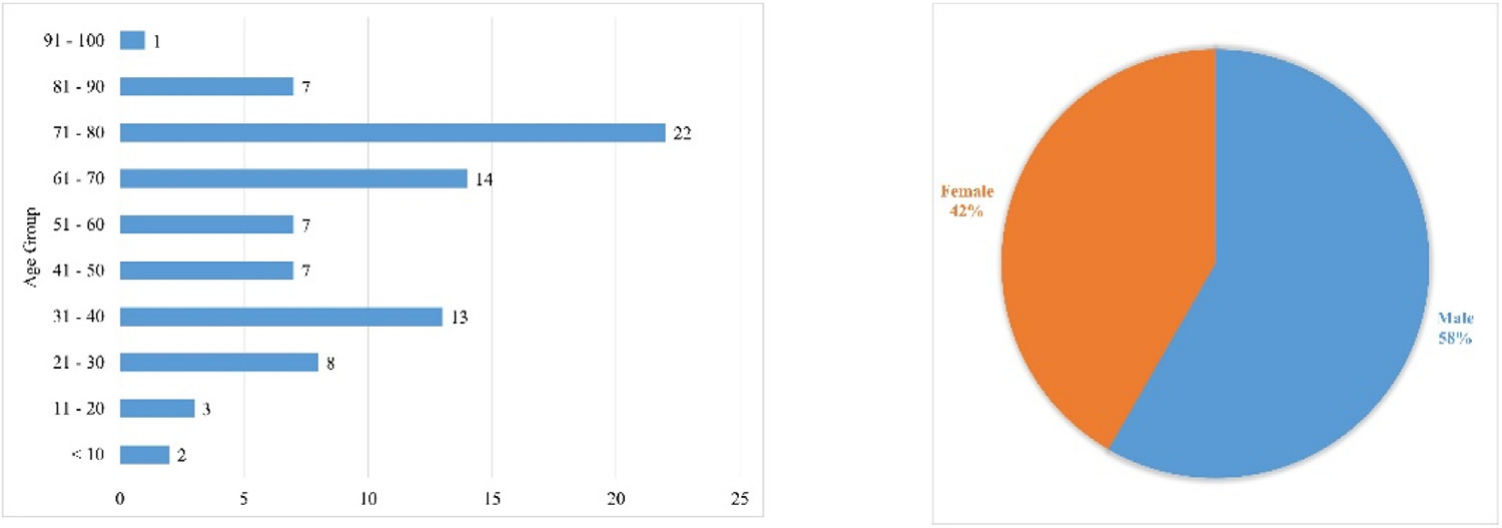
Distribution of demographic characteristics concerning age and gender among the KAUHC's studies.

An overview of the dataset demographics is presented in Fig 6. The WCE studies were retrospectively collected from 157 patients at King Abdulaziz University Hospital between December 2019 and December 2023, where 58 % and 42 % of studies were recorded from male and female patients, respectively. The majority of patients were between age range of 71–80 year old (*n* = 22), 61–70 year-old (*n* = 14), and 31–40 year-old (*n* = 13).

## 4 Experimental Design, Materials and Methods

This section provides a detailed description of the tool employed for data collection and the evaluation methods used to verify the annotation process of assigning the pictures to the relevant class.

### 4.1 OMOM WCE

**Fig. 7:**
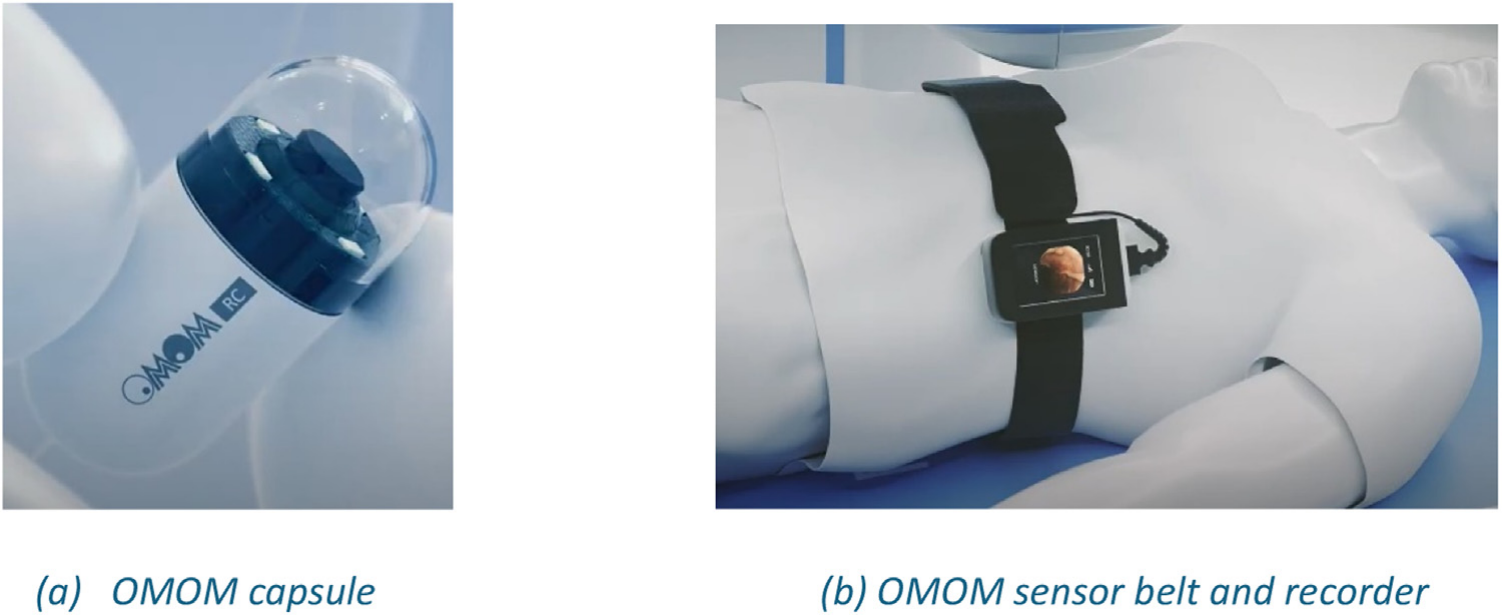
Overview of the OMOM Capsule System [7].

The studies were recorded through the OMOM WCE system [9]. Fig 7 portrays an overview of the system used to collect the WCE studies. As with any typical WCE system, OMOM WCE is a tubular-shaped camera used to thoroughly view the mucosa^2^ inside the GI tract. The capsule itself, a sensor belt with a receiver, and a workstation for downloading and analyzing images are the three main parts of a capsule system [10]. The OMOM capsule is a device measuring 28 mm in length 13 mm in diameter and is characterized by its lightweight design. It provides frame rates of 0.5, 1, or 2 frames per second and a wide viewing angle of 140°. The capsule sends compressed images via an integrated antenna to a sensor array that can be worn by the patient and integrated into a sensor belt or vest. Subsequently, the array connects to a portable storage device for real-time image display. This storage device interfaces with a computer for data transfer and analysis. Notably, these capsules do not store data independently, ensuring their safe disposal after excretion [3].

### 4.2 Data collection and reprocessing

**Fig. 8:**
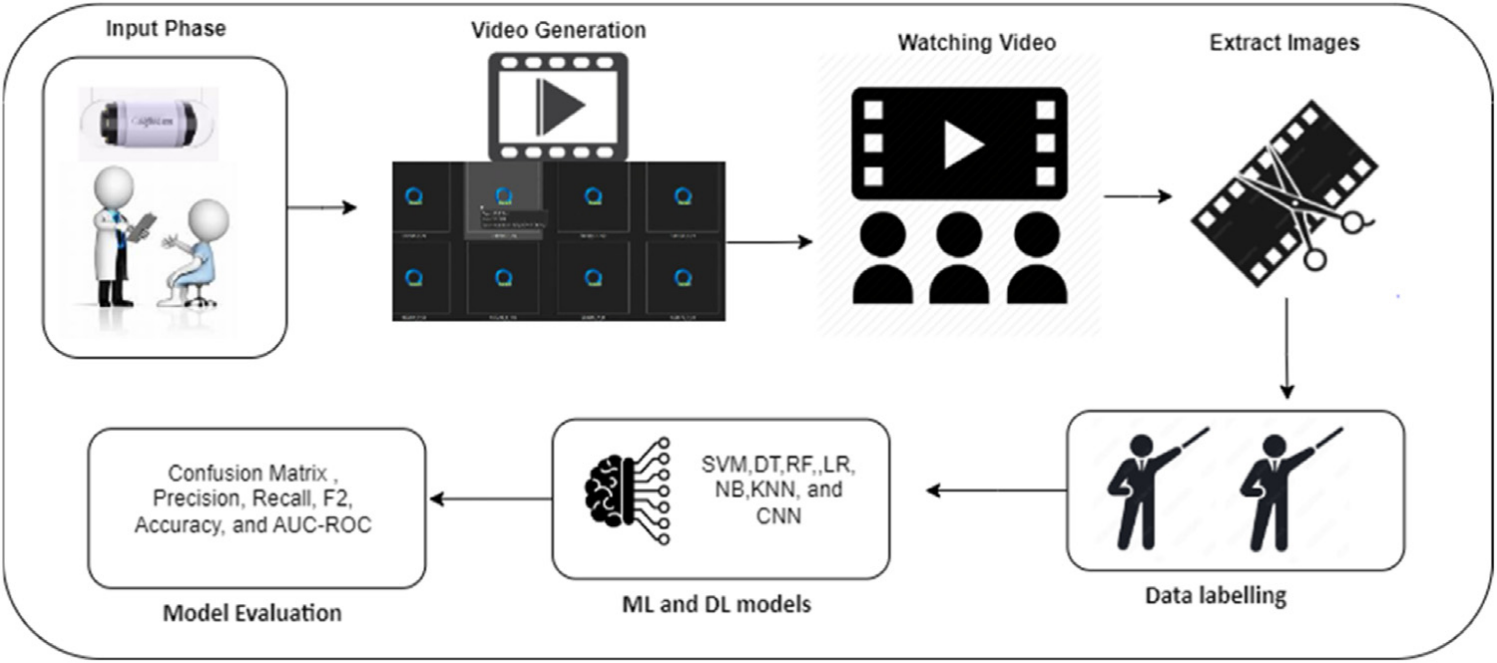
: Overview of the KAUHC dataset collection process.

Fig 8 indicates an overview of the KAUHC dataset collection process. The collection process entails seven phases. Initially, the process commences with patient admission, preparation for GI tract screening, and the ingestion of the OMOM capsule. The second phase involves the capture, wireless transmission, and recording of a sequence of videos using the OMOM system. Then, a gastroenterologist conducts a manual review of the recorded videos in order to not only identify a point of interest (POI) but also choose the most relevant frames in terms of pathological abnormalities. Subsequently, the POI frames are exported, labeled, and submitted for the validation phase. These annotated frames serve as training and testing sets and are utilized as input for ML classification models. Finally, the evaluation metrics were applied to assess the study's outcomes.

#### 4.2.1 WCE studies acquisition

The patients scheduled for WCE were directed to undergo bowel preparation^3^ the day prior to the procedure and to fast overnight (8–12 h). On the procedure day, patients ingested a small capsule, followed by permission to consume a light breakfast after three hours and a light meal after five hours.

The capsule traverses the SB propelled by peristaltic movements,^4^ capturing images at a fixed frame rate during the capsule's journey through the GI tract. These images are transmitted to a data recorder carried on a belt outside the patient's body. The following day, patients return to the endoscopy unit for data and image retrieval. Subsequently, the capsule is naturally expelled in the patient's stool within 24–48 h.

**Fig. 9:**
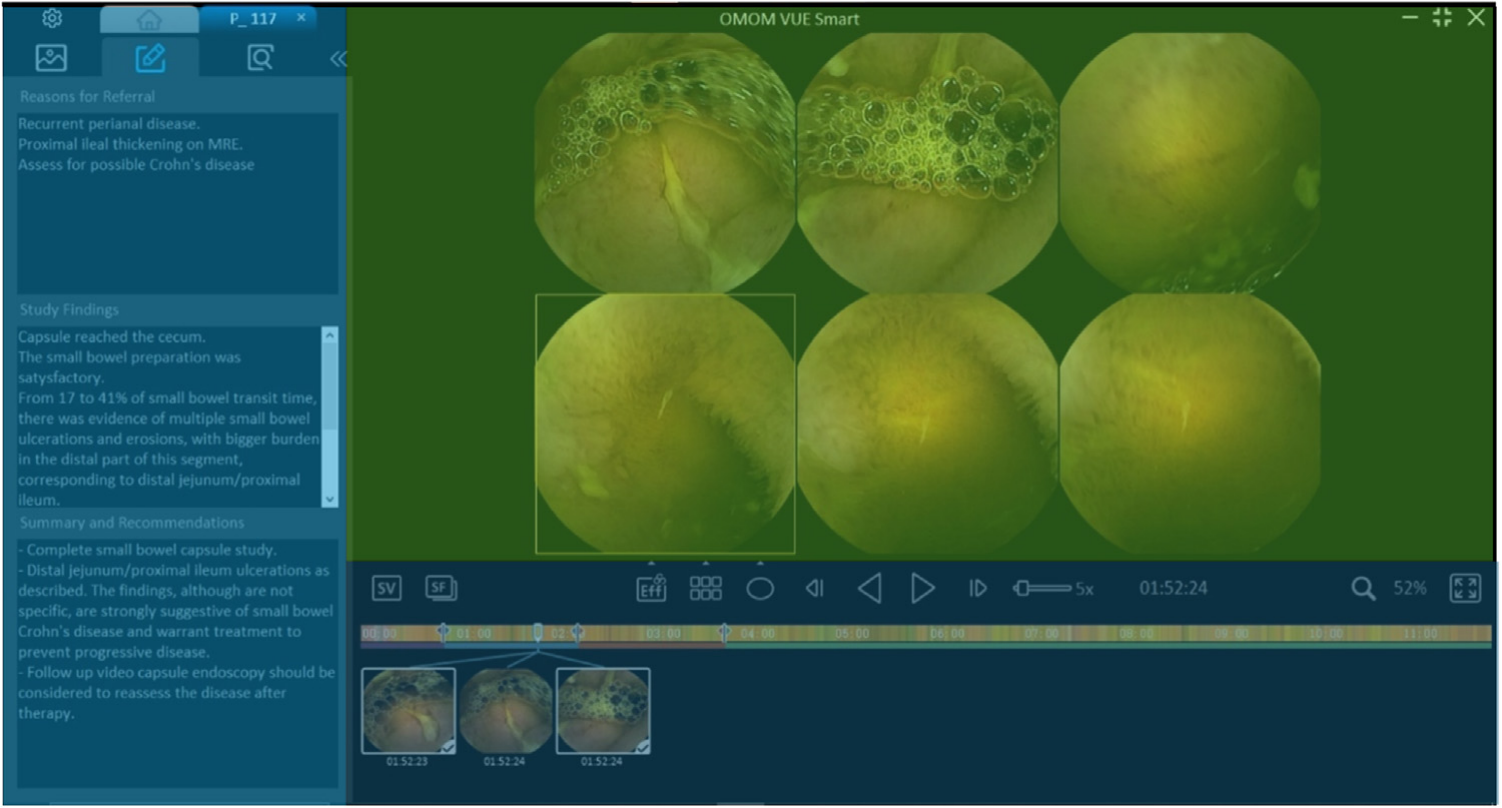
A sample of the WCE study is shown through OMOM VUE Smart software. Each study entails selected WCE frames with pathological abnormalities and the gastroenterologists’ insights. The left side: gastroenterologists’ insights in terms of the reason for referral, WCE study outcomes, and the WCE study summary and recommendations. The right-up side: the WCE video stream and viewing area for gastroenterologists. The right-bottom side: a set of notation tools and the exported SB frames.

Senior gastroenterologists review the capsule endoscopy video, calculate the average transit time of the WCE in the stomach and small intestine, and identify anatomical landmarks. In addition to identifying anatomical features and calculating the average transit time of the WCE in the stomach and small intestine, it is included. Subsequently, gastroenterologists generated a WCE examination report containing annotated frames to emphasize detected anomalies. The capsule recording, gastroenterologists’ chosen frames, and the examination report are consolidated into an individual study file known as ‘.vue’, as shown in Fig 9.

#### 4.2.2 Dataset de-identification and labeling

Before accessing the original studies, personal identification details of patients (such as names, addresses, and phone numbers) were anonymized prior to retrieval. The OMOM VUE Smart software, developed by OMOM, was utilized to open the exclusive study files saved in a specific data format (i.e., ‘.vue’ format) sourced from the Data Management Office repository at KAUH. Subsequent to the de-identification procedure, the studies were retained in the proprietary video format. The OMOM VUE Smart software offers annotation functionalities, enabling gastroenterologists to capture and label specific frames of interest. WCE studies were categorized into three groups: normal, AVM, and ulcer. gastroenterologists were responsible for identifying the SB region and pinpointing pertinent frames within the studies. Quality assurance measures involved three gastroenterologists conducting the studies, with the first and second specialists reviewing and identifying relevant frames and the third physician validating the initial findings.

#### 4.2.3 Inclusion and exclusion criteria

The selection was restricted to completed WCE studies that satisfied the requirements of patient-initiated capsule ingestion and successful SB traversal. Studies on WCE that did not adequately prepare the bowels were excluded. WCE studies with thorough documentation and consensus on investigations that allowed for the sequential labeling of pictures showing anatomical structures and pathological results met the inclusion criteria.

### 4.3 Evaluation

This section describes the evaluation methods used to verify the annotation process of assigning the pictures to the relevant class. In this dataset, three methods were utilized: Cohen's Kappa, three medical experts, and Model-Based Evaluation.

#### 4.3.1 Cohen's kappa

Cohen's Kappa (κ) is a statistical model that measures the agreement between the raters (judges). The result of K is the value between 0 and 1; if this value is higher, it indicates a high agreement between the raters (judges). K is calculated using the following mathematical formula.

(1) $$K=\;\frac{P_{o}-\;P_{e}}{1-\;P_{e}}$$

Where

(2) $$p_{o}=\frac{\;\text{agreement}\;\text{between}\;\text{two}\;\text{raters}\;\text{in}\;\text{classify}\;\text{images}\;\text{in}\;\text{both}\;\text{classes}}{\text{total}\;\text{number}\;\text{of}\;\text{images}\;\text{in}\;\text{the}\;\text{dataset}}$$

(3) $$p_{e=P\left(\text{Yes}\right)+P\left(\text{No}\right)}$$

(4) $$P\left(\;\text{Yes}\right)=\;\frac{\text{Number}\;\text{of}\;\text{rater}\;1\;\text{said}\;\text{Yes}\;}{\text{Total}\;\text{number}\;\text{of}\;\text{responses}\;}\;X\;\frac{\text{Number}\;\text{of}\;\text{rater}\;2\;\text{said}\;\text{Yes}}{\text{Total}\;\text{number}\;\text{of}\;\text{responses}\;}$$

(5) $$P\left(\;\text{No}\right)=\;\frac{\text{Number}\;\text{of}\;\text{rater}\;1\;\text{said}\;\text{No}\;}{\text{Total}\;\text{number}\;\text{of}\;\text{responses}\;}\;X\;\frac{\text{Number}\;\text{of}\;\text{rater}\;2\;\text{said}\;\text{No}}{\text{Total}\;\text{number}\;\text{of}\;\text{responses}\;}$$

The proportion agreement between judges is $P_{o}$ while the expected agreement proportion by chance is $P_{e}$. As a result, the agreement between the two raters for dataset 1 reached to K is 81 %, which indicates a perfect agreement between the judges (raters) while dataset 2 reached 83 and dataset 3 reached 80. Lastly, Table 1 provides information about the size of the final dataset.

**Table 1:** Dataset description.

No.	Class name	Class size
1	AVM	673
2	Normal	2156
3	Ulcer	472

#### 4.3.2 Three medical experts

Three medical experts in the same medical field helped annotate the datasets into categories known as classes (predefined labels). The criterion is that if two annotators agree and assign the picture to the same categories, the decision will be made. Table 2 represents the annotations of the three medical experts.

**Table 2:** Example of annotation criteria.

Item(s)	MEA 1	MEA 2	MEA2	Final Results
Image1	✓	✓	✓	✓
Image2	✓	✗	✓	✓
Image3	✓	✗	✗	✗
Image4	✗	✗	✗	✗

#### 4.3.3 Model-based evaluation

This section explains the explains the measurements, experiment settings and the results of the experiments. All experiments were conducted based on the two methods of annotation for the three datasets.

##### 4.3.3.1 Model performance measurements

All the experiments that were conducted in this study to validate the correctness of the annotation process used the most common metrics. These common evaluation metrics used consisted of Precision, Recall, Accuracy, and F1 score. Accuracy tells us what percentage of images classified as generated are actually generated, as shown in the following equation.

(6) $$\text{Accuracy}=\;\frac{\text{Number}\;\text{of}\;\text{the}\;\text{correct}\;\text{images}\;\text{classified}\;}{\text{Total}\;\text{number}\;\text{of}\;\text{images}}$$

Precision measures the ratio of true positives (TP) to the sum of true positives and false positives (FP). Precision tells us what percentage of images are classified as were actually generated as shown in the following equation.

(7) $$\text{Precison}=\;\frac{\text{Number}\;\text{of}\;\text{the}\;\text{correct}\;\text{images}\;\text{classified}\;}{\text{Total}\;\text{number}\;\text{of}\;\text{relevant}\;\text{images}}$$

The recall is the ratio of true positives to the sum of true positives and false negatives. It tells us the percentage of generated images that were correctly identified as such. It is represented in the following equation

(8) $$\text{Recall}=\;\frac{\text{Number}\;\text{of}\;\text{the}\;\text{correct}\;\text{images}\;\text{classified}\;}{\text{Total}\;\text{number}\;\text{of}\;\text{images}\;\text{classified}\;}$$

This is the harmonic mean of precision and recall calculated using the F1 score as represented in (9). The AUC-ROC level is calculated by plotting the true positive rate (TPR) against the false positive rate (FPR).

(9) $$F1-\text{Score}=2\;X\;\frac{\text{Precision}+\text{Recall}}{\text{Precision}\;X\;\text{Recall}}$$

##### 4.3.3.2 Experiments settings

In all of the experiments, the “Google Collaborative lab” was used to utilize the GPU environment. Python libraries were used for the ML experiments, and the “sklearn” library was used to import ML classifiers and split the datasets for training and testing. “Matplotlib” and “seaborn” libraries were used to visualize the confusion matrix. In addition, for image loading and preprocessing, “tensorflow” and “keras”, as well as “preprocessing.imag” have been used. The machine learning classifiers’ hyperparameters are as shown in Table 3. It is also to be mentioned that the datasets for all the experiments have been divided into two parts, 80 % for training and 20 % for testing.

**Table 3:** ML hyperparameters.

Classifier	Default parameters
NB	No specific default parameters to set
KNN	n_neighbors=5, weights='uniform', algorithm='auto', leaf_size=30, *p* = 2 (Euclidean distance)
LR	penalty='l2′, dual=False, tol=1e-4, C = 1.0,, max_iter=100, multi_class='auto',
SVM	C = 1.0, kernel='rbf', degree=3, gamma='scale', coef0=0.0, shrinking=True,, tol=1e-3, cache_size=200
DT	criterion='gini', splitter='best', max_depth=None, min_samples_split=2, min_samples_leaf=1
RF	n_estimators=100, criterion='gini', max_depth=None, min_samples_split=2, min_samples_leaf=1, min_weight_fraction_leaf=0.0, max_features='auto', bootstrap=True

##### 4.3.3.3 Experiments results

The proposed dataset was evaluated using three types of scenarios to verify the correctness of the annotation process in classifying the images. These methods are model based. The three types are; dataset1, which is Normal and AVM, dataset2, which is Normal and Ulcer, and Dataset 3, which is Normal, AVM, and Ulcer. The descriptions of the datasets are shown in Table 1. The experiment was conducted using six commonly used machine learning classifiers, which specifically are Decision Tree (DT), Random Forest (RF), K-Nearest Neighbors (KNN), Logistic Regression (LR), Naive Bayes (NB), and Support Vector Machine (SVM). All ML classifiers were measured on Precision, Recall, F1, Accuracy, and AUC-ROC. Each of the experiments was conducted twice, once without using SMOT and the other while using SMOT.

In the first scenario, the experiments were conducted using the first dataset, which consisted of binary classification of normal and AVM classes. In this scenario two types of experiments were conducted due to the imbalanced dataset. The first experiment was conducted without SMOT, while the second experiment was conducted with SMOT. In both of the experiments, the accuracy, F1-score, recall, and precision show high records. Table 4, Table 5 both show the comparison between the ML classifiers with SMOT and without SMOT, respectively.

**Table 4:** Comparison between Precision, recall, F1, and Accuracy of experiments without SMOT for dataset 1.

ML Classifiers	Class/Average	Precision	Recall	F1-Score	Accuracy
DT	AVM	97.86 %	97.16 %	97.51 %	98.76 %
	Normal	99.06 %	99.29 %	99.18 %	
	Average	98.46 %	98.23 %	98.34 %	
KNN	AVM	100.00 %	75.89 %	86.29 %	93.99 %
	Normal	92.59 %	100.00 %	96.15 %	
	Average	96.30 %	87.94 %	91.22 %	
LR	AVM	100.00 %	92.91 %	96.32 %	98.23 %
	Normal	97.70 %	100.00 %	98.84 %	
	Average	98.85 %	96.45 %	97.58 %	
NB	AVM	61.94 %	58.87 %	60.36 %	80.74 %
	Normal	86.57 %	88.00 %	87.28 %	
	Average	74.26 %	73.43 %	73.82 %	
RF	AVM	100.00 %	88.65 %	93.98 %	97.17 %
	Normal	96.37 %	100.00 %	98.15 %	
	Average	98.19 %	94.33 %	96.07 %	
SVM	AVM	100.00 %	81.56 %	89.84 %	95.40 %
	Normal	94.24 %	100.00 %	97.03 %	
	Average	97.12 %	90.78 %	93.44 %

**Table 5:** Comparison between Precision, recall, F1, and Accuracy of experiments with SMOT for dataset 1.

ML Classifiers	Class	Precision	Recall	F1-Score	Accuracy
DT	AVM	100.00 %	100.00 %	100.00 %	100.00 %
	Normal	100.00 %	100.00 %	100.00 %	
	Average	100.00 %	100.00 %	100.00 %	
KNN	AVM	83.02 %	93.62 %	88.00 %	93.63 %
	Normal	97.79 %	93.65 %	95.67 %	
	Average	90.40 %	93.63 %	91.84 %	
LR	AVM	100.00 %	94.33 %	97.08 %	98.58 %
	Normal	98.15 %	100.00 %	99.07 %	
	Average	99.08 %	97.16 %	98.07 %	
NB	AVM	62.22 %	59.57 %	60.87 %	80.91 %
	Normal	86.77 %	88.00 %	87.38 %	
	Average	74.50 %	73.79 %	74.13 %	
RF	AVM	96.90 %	88.65 %	92.59 %	96.46 %
	Normal	96.34 %	99.06 %	97.68 %	
	Average	96.62 %	93.86 %	95.14 %	
SVM	AVM	98.46 %	90.78 %	94.46 %	97.34 %
	Normal	97.02 %	99.53 %	98.26 %	
	Average	97.74 %	95.15 %	96.36 %	

**Fig. 10:**
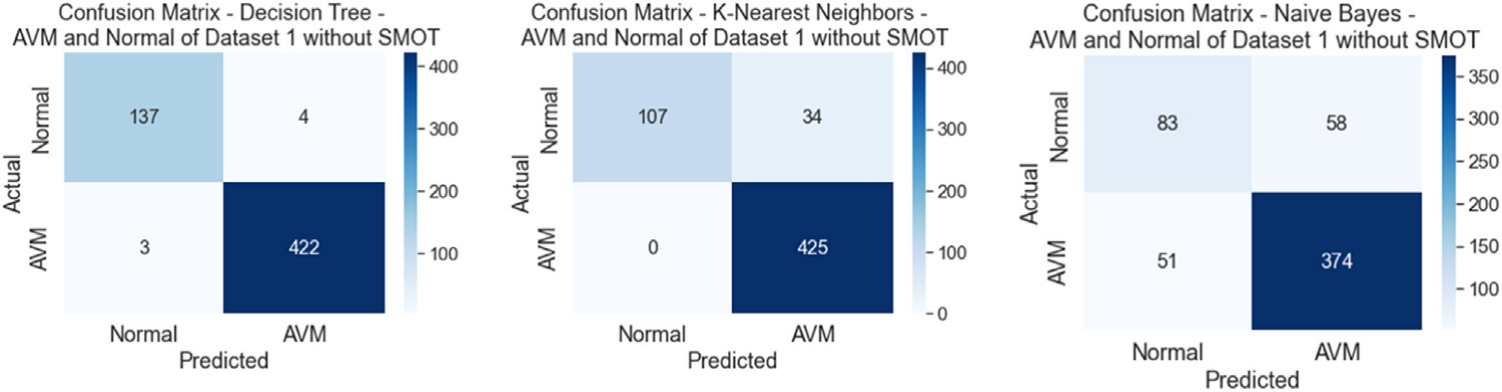
Confusion matrix of DT, KNN, and NB for experiments without SMOT of dataset 1.

**Fig. 11:**
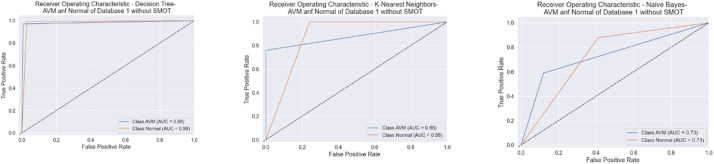
AUC-ROC of DT, KNN, and NB for experiments without SMOT of dataset 1.

**Fig. 12:**
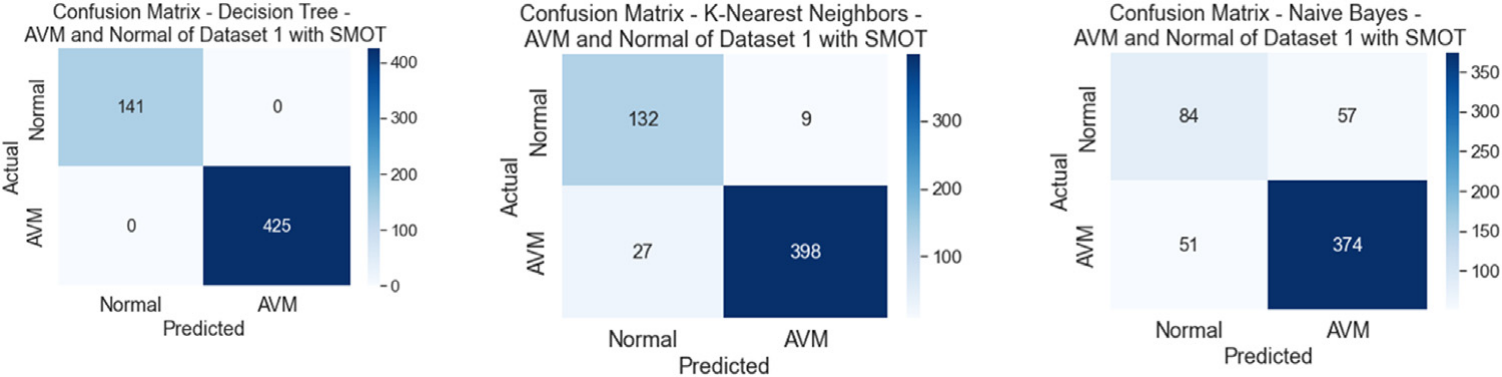
Confusion matrix of DT, KNN, and NB for experiments with SMOT of dataset 1.

**Fig. 13:**
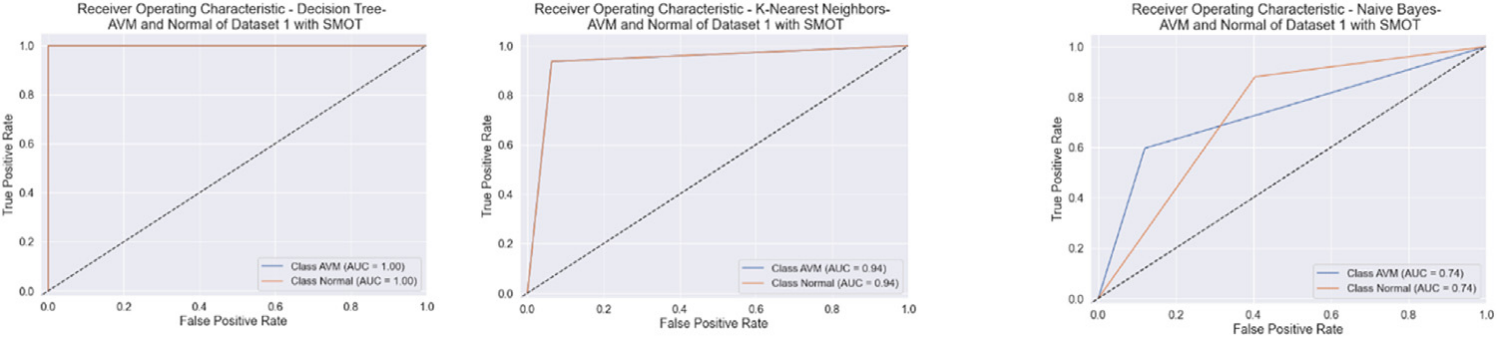
AUC-ROC of DT, KNN, and NB for experiments with SMOT of dataset 1.

All six classifiers in the first experiment (without SMOT) reached high accuracies; all reached accuracy scores above 90 %, except for NB, which reached an accuracy score of 80 %. From the literature, NB is known to often have low performance compared to the other ML classifiers. In the second experiment (with SMOT), the same trend can be seen with all the ML classifiers, with all the classifiers reaching high scores above 90 % except NB, which records 80 % in both experiments. Generally, this indicates that the annotation process of the two classes performed well, as shown in the confusion matrix and AUC-ROC in Fig. 10, Fig. 11, Fig. 12, Fig. 13.

In the second scenario, the experiments were conducted using the second dataset, which consisted of binary classifications of normal and ulcer classes. As in the first scenario, two types of experiments were conducted due to the imbalanced dataset (one with SMOT and the other without SMOT). This experiment of the annotation process for the second dataset was verified using the ML mentioned above classifiers. Table 6, Table 7 show the precision, recall, f-measure, and accuracy for both of the conducted experiments, with SMOT and without SMOT.

**Table 6:** Comparison between Precision, Recall, F1, and Accuracy of experiments without SMOT for dataset 2.

ML Classifiers	Class	Precision	Recall	F1-Score	Accuracy
DT	Normal	100.00 %	100.00 %	100.00 %	100.00 %
	Ulcer	100.00 %	100.00 %	100.00 %	
	Average	100.00 %	100.00 %	100.00 %	
KNN	Normal	95.19 %	98.42 %	96.77 %	94.48 %
	Ulcer	89.86 %	73.81 %	81.05 %	
	Average	92.52 %	86.11 %	88.91 %	
LR	Normal	99.55 %	100.00 %	99.77 %	99.61 %
	Ulcer	100.00 %	97.62 %	98.80 %	
	Average	99.77 %	98.81 %	99.28 %	
NB	Normal	93.71 %	74.21 %	82.83 %	74.14 %
	Ulcer	35.23 %	73.81 %	47.69 %	
	Average	64.47 %	74.01 %	65.26 %	
RF	Normal	96.92 %	99.77 %	98.33 %	97.14 %
	Ulcer	98.59 %	83.33 %	90.32 %	
	Average	97.76 %	91.55 %	94.33 %	
SVM	Normal	97.11 %	98.87 %	97.98 %	96.57 %
	Ulcer	93.42 %	84.52 %	88.75 %	
	Average	95.27 %	91.70 %	93.37 %

**Table 7:** Comparison between Precision, Recall, F1, and Accuracy of experiments with SMOT for dataset 2.

ML Classifiers	Class	Precision	Recall	F1-Score	Accuracy
DT	Normal	98.66 %	99.77 %	99.21 %	98.66 %
	Ulcer	98.73 %	92.86 %	95.71 %	
	Average	98.70 %	96.32 %	97.46 %	
KNN	Normal	99.25 %	90.27 %	94.55 %	91.25 %
	Ulcer	65.32 %	96.43 %	77.88 %	
	Average	82.29 %	93.35 %	86.22 %	
LR	Normal	99.55 %	100.00 %	99.77 %	99.61 %
	Ulcer	100.00 %	97.62 %	98.80 %	
	Average	99.77 %	98.81 %	99.28 %	
NB	Normal	94.87 %	75.34 %	83.98 %	75.85 %
	Ulcer	37.71 %	78.57 %	50.97 %	
	Average	66.29 %	76.96 %	67.48 %	
RF	Normal	98.66 %	100.00 %	99.33 %	98.85 %
	Ulcer	100.00 %	92.86 %	96.30 %	
	Average	99.33 %	96.43 %	97.81 %	
SVM	Normal	99.31 %	97.51 %	98.40 %	97.33 %
	Ulcer	88.04 %	96.43 %	92.05 %	
	Average	93.68 %	96.97 %	95.22 %	

Similarly to the first scenario, In the second scenario, all of the six classifiers in the first experiment (without SMOT) reached high accuracies; all reached accuracy scores above 90 % except for NB, which reached an accuracy score of 70 %. In the second experiment (with SMOT), all of the ML classifiers also reached accuracy scores higher than 90 %, except for NB, which reached 70 % as well. In addition, LR also notably reaches the highest accuracy score. All are shown in Table 5 (without SMOT) and Table 6 (with SMOT).

**Fig. 14:**
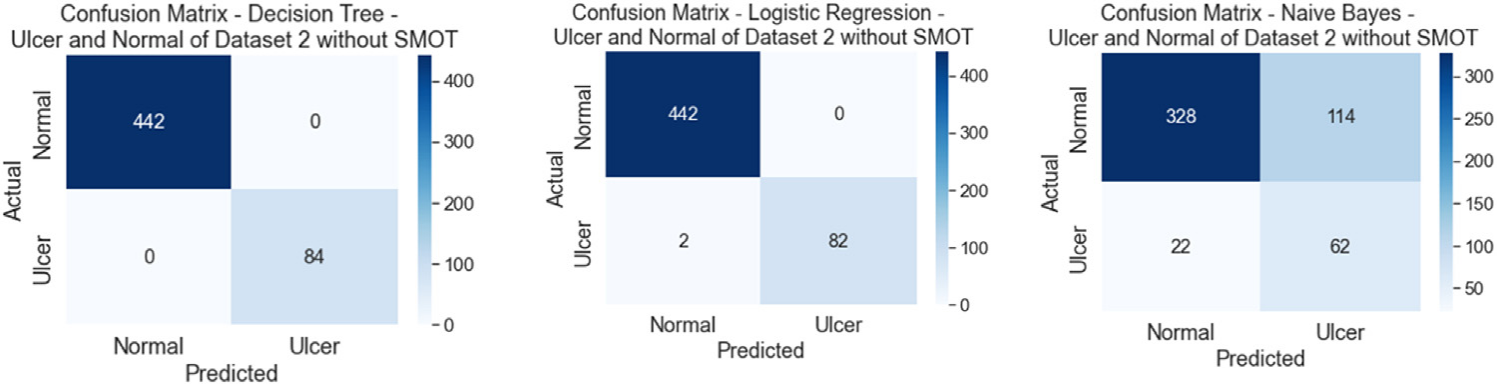
Confusion matrix of DT, LR, and NB for experiments without SMOT of dataset 2.

**Fig. 15:**
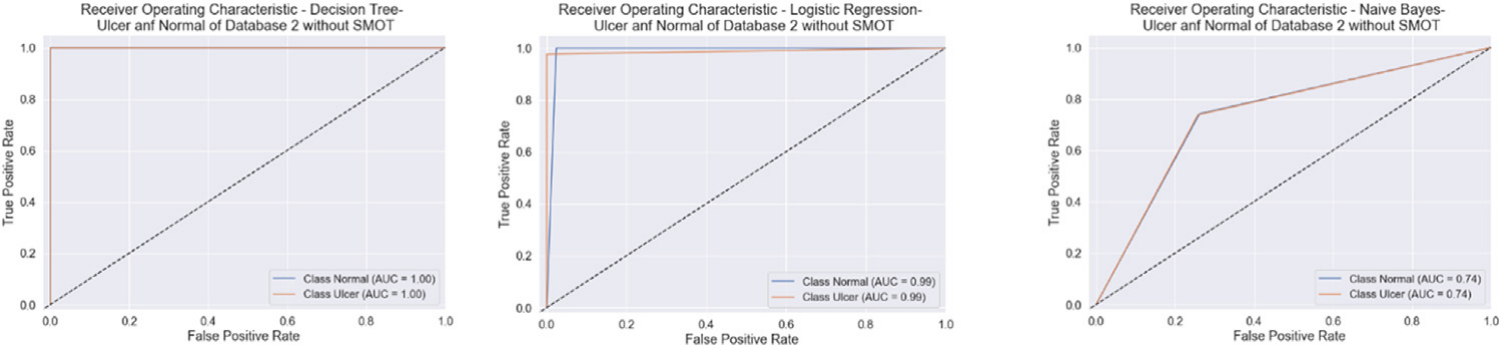
AUC-ROC of DT, LR, and NB for experiments without SMOT of dataset 2.

**Fig. 16:**
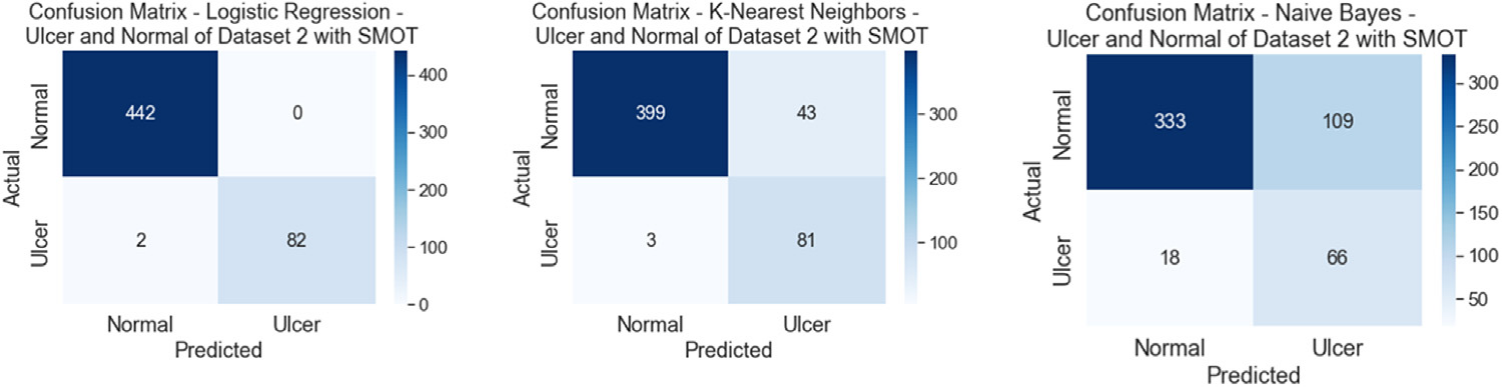
Confusion matrix of LR, KNN, and NB for experiments with SMOT of dataset 2.

**Fig. 17:**
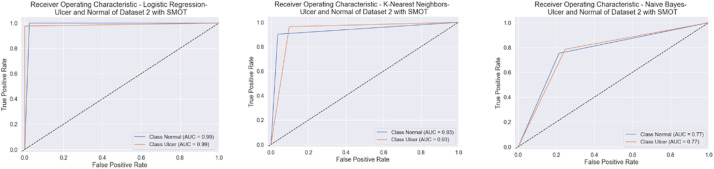
AUC-ROC of LR, KNN, and NB for experiments with SMOT of dataset 2.

The precision, recall, F-Measure, and accuracy between the experiments (with and without SMOT) are shown in Table 5, Table 6, respectively. This indicates that the annotation process of the two classes performed well, as shown in the confusion matrix and AUC-ROC in Fig. 14, Fig. 15, Fig. 16, Fig. 17.

In the third scenario, the experiments were conducted using the third dataset, which consisted of three classes: AVM, Normal, and Ulcer. As in the previous scenarios, two types of experiments were conducted in this scenario due to the imbalanced dataset (one with SMOT and the other without SMOT). This experiment of the annotation process for the second dataset was verified using the ML mentioned above classifiers. Table 7, Table 8 show the precision, recall, f-measure, and accuracy for both of the conducted experiments, with SMOT and without SMOT.

**Table 8:** Comparison between Precision, Recall, F1, and Accuracy of experiments without SMOT for dataset 3.

ML Classifiers	Class	Precision	Recall	F1-Score	Accuracy
DT	AVM	97.48 %	98.10 %	97.79 %	98.94 %
	Normal	99.76 %	99.04 %	99.40 %	
	Ulcer	97.75 %	100.00 %	98.86 %	
	Average	98.33 %	99.05 %	98.68 %	
KNN	AVM	84.12 %	90.51 %	87.20 %	87.44 %
	Normal	95.21 %	86.06 %	90.40 %	
	Ulcer	66.96 %	88.51 %	76.24 %	
	Average	82.10 %	88.36 %	84.61 %	
LR	AVM	100.00 %	94.94 %	97.40 %	98.63 %
	Normal	98.81 %	100.00 %	99.40 %	
	Ulcer	95.56 %	98.85 %	97.18 %	
	Average	98.12 %	97.93 %	97.99 %	
NB	AVM	55.56 %	53.80 %	54.66 %	57.33 %
	Normal	73.23 %	57.21 %	64.24 %	
	Ulcer	30.60 %	64.37 %	41.48 %	
	Average	53.13 %	58.46 %	53.46 %	
RF	AVM	99.31 %	90.51 %	94.70 %	96.06 %
	Normal	94.32 %	99.76 %	96.96 %	
	Ulcer	100.00 %	88.51 %	93.90 %	
	Average	97.87 %	92.92 %	95.19 %	
SVM	AVM	100.00 %	90.51 %	95.02 %	95.76 %
	Normal	94.70 %	98.80 %	96.71 %	
	Ulcer	94.05 %	90.80 %	92.40 %	
	Average	96.25 %	93.37 %	94.71 %	

The majority of the six classifiers in the first experiment (without SMOT) reached high accuracies, above 90 %, except for NB and KNN, with NB attaining a low accuracy score of 50 % and KNN achieving an accuracy score less than 90 % being 80 %. In the second experiment (with SMOT), the result was split in half. Half of the classifiers achieved an accuracy of under 90 % (NB, SVM, and KNN), and the other half achieved higher than 90 % (DT, LR, and RF). It is to be noted that although SVM received a lower score than 90 %, it still performed desirably and achieved an accuracy of 89 %, which makes SVM the best-performing classifier, with an accuracy of under 90 %. Similarly to the previous scenarios, the lowest accuracy was from NB, which had an accuracy of 50 %. All are shown in Tables 8 (without SMOT) and 9 (with SMOT).

**Fig. 18:**
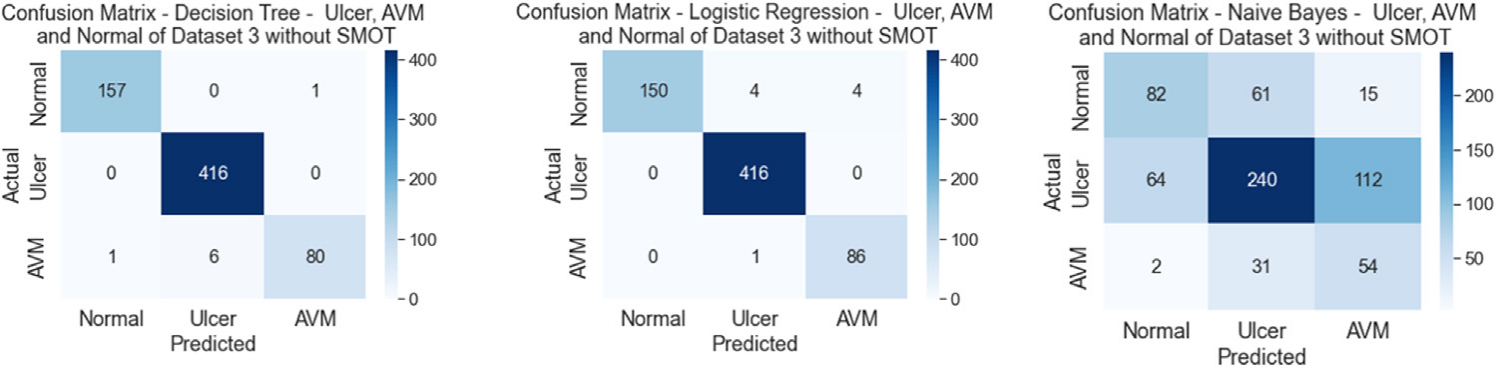
Confusion matrix of DT, LR and NB for experiments without SMOT of dataset 3.

**Fig. 19:**
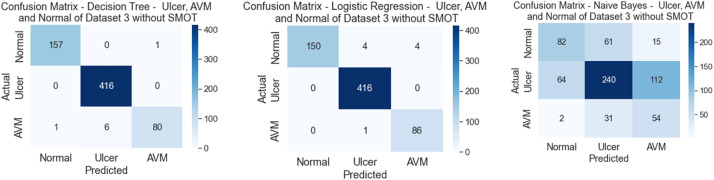
Confusion matrix of DT, LR, and NB for experiments with SMOT of dataset 3.

The precision, recall, F-Measure, and accuracy between the experiments (with and without SMOT) are shown in Table 8, Table 9, respectively. This proves that the classifiers performed desirably by classifying the relevant images, and this can be seen from the aforementioned three classes, as shown in the confusion matrix in Fig. 18, Fig. 19.

**Table 9:** Comparison between Precision, Recall, F1, and Accuracy of experiments with SMOT for dataset 3.

	Class	Precision	Recall	F1-Score	Accuracy
DT	AVM	99.37 %	99.37 %	99.37 %	98.78 %
	Normal	98.58 %	100.00 %	99.28 %	
	Ulcer	98.77 %	91.95 %	95.24 %	
	Average	98.90 %	97.11 %	97.96 %	
KNN	AVM	100.00 %	73.42 %	84.67 %	88.04 %
	Normal	85.24 %	98.56 %	91.42 %	
	Ulcer	87.50 %	64.37 %	74.17 %	
	Average	90.91 %	78.78 %	83.42 %	
LR	AVM	100.00 %	94.94 %	97.40 %	98.63 %
	Normal	98.81 %	100.00 %	99.40 %	
	Ulcer	95.56 %	98.85 %	97.18 %	
	Average	98.12 %	97.93 %	97.99 %	
NB	AVM	55.41 %	51.90 %	53.59 %	56.88 %
	Normal	72.29 %	57.69 %	64.17 %	
	Ulcer	29.83 %	62.07 %	40.30 %	
	Average	52.51 %	57.22 %	52.69 %	
RF	AVM	100.00 %	82.91 %	90.66 %	92.27 %
	Normal	89.08 %	100.00 %	94.22 %	
	Ulcer	98.41 %	71.26 %	82.67 %	
	Average	95.83 %	84.73 %	89.18 %	
SVM	AVM	100.00 %	74.68 %	85.51 %	89.25 %
	Normal	85.86 %	99.28 %	92.08 %	
	Ulcer	95.16 %	67.82 %	79.19 %	
	Average	93.67 %	80.59 %	85.60 %

Overall, based on the experimental results it shows that the annotation process has been performed correctly in classifying the images into the relevant classes using the methods of Cohenʼs Kappa measurements and the annotation by the medical experts, and this was verified by using a model-based strategy.

## Limitations

Several obstacles persist throughout the data collection process in light of the capacity of WCE datasets, including KAUHC, to prompt the detection of pathological abnormalities in SB examinations. Firstly, a significant challenge associated with these datasets is the considerable time required to interpret WCE studies. A definitive standard for interpreting WCE findings is absent, potentially resulting in an indeterminate misinterpretation rate. In addition, the KAUHC dataset was curated retrospectively, introducing the potential for selection bias whereby the examined sample may not be as representative as desired. As a result, participating in numerous validation phases and incorporating more gastroenterologists were involved, potentially alleviating these challenges.

Furthermore, inadequate visualization of GI landmarks often arises due to the rapid transit of the capsule without effective monitoring and technical limitations concerning frame rate and viewing angles. Pathological abnormalities might only present in a few images, potentially evading physician identification or the risk of oversights of these abnormalities. To address these challenges, particular studies underwent reassessment, with inclusion criteria focused on accuracy and high-resolution frames. OMOM VUE Smart software, equipped with enhanced functionalities for precise annotation and exporting of all abnormalities, was also employed.

## Ethics Statement

The research adhered to the principles stated in the KAU ethics committee regulations. Written informed consent for the complete capsule endoscopy procedure was obtained from all patients. Data collection was carried out while upholding the confidentiality of patient data. Furthermore, any information disseminated to external entities must undergo comprehensive de-identification processes.

## CRediT authorship contribution statement

**Hamza Ghandorh:** Conceptualization, Methodology, Data curation, Investigation, Validation, Writing – original draft. **Hamza H. Bali:** Investigation, Data curation, Writing – original draft. **Wael M.S. Yafooz:** Methodology, Software, Validation, Writing – original draft. **Wadii Boulila:** Methodology, Resources, Writing – review & editing. **Majid Alsahafi:** Conceptualization, Resources, Supervision.

## Footnotes

1 Wireless or Video Capsule Endoscopy (VCE) or Small-bowel Capsule Endoscopy (SB-CE) terms might be used in this manuscript interchangeably

2 The mucosa layer, the innermost layer, is responsible for absorption and secretion of nutrients with GI tract

3 Bowel preparation procedure entails the administering of polyethylene glycol electrolyte powder orally with one liter of drinking water for small-bowel cleansing.

4 Peristalsis involves the involuntary contraction and relaxation of longitudinal and circular muscles along the digestive tract [11].
